# Histopathologic tumor invasion of superior mesenteric vein/ portal vein is a poor prognostic indicator in patients with pancreatic ductal adenocarcinoma: results from a systematic review and meta-analysis

**DOI:** 10.18632/oncotarget.15938

**Published:** 2017-03-06

**Authors:** Ailin Song, Farong Liu, Lupeng Wu, Xiaoying Si, Yanming Zhou

**Affiliations:** ^1^ Department of General Surgery, Second Hospital of Lanzhou University, Lanzhou, China; ^2^ Department of Hepatobiliary and Pancreatovascular Surgery, First affiliated Hospital of Xiamen University, Xiamen, China

**Keywords:** pancreatic adenocarcinoma, survival, prognosis, superior mesenteric vein, portal vein

## Abstract

**Background:**

The impact of histopathologic tumor invasion of the superior mesenteric vein (SMV)/portal vein (PV) on prognosis in patients with pancreatic ductal adenocarcinoma (PDAC) after pancreatectomy remains controversial. A meta-analysis was performed to assess this issue.

**Results:**

Eighteen observational studies comprising 5242 patients were eligible, of whom 2199 (41.9%) patients received SMV/PV resection. Histopathologic tumor invasion was detected in 1218 (58.1%) of the 2096 resected SMV/PV specimens. SMV/PV invasion was associated with higher rates of poor tumor differentiation (*P* = 0.002), lymph node metastasis (*P* < 0.001), perineural invasion (*P* < 0.001), positive resection margins (*P* = 0.004), and postoperative tumor recurrence (*P* < 0.001). SMV/PV invasion showed a significantly negative effect on survival in total patients who underwent pancreatectomy with and without SMV/PV resection (hazard ratio [HR]: 1.21, 95% confidence interval [CI]: 1.08–1.35; *P* = 0.001) and in patients who underwent pancreatectomy with SMV/PV resection (HR: 1.88, 95% CI, 1.48–2.39; *P* < 0.001).

**Materials And Methods:**

A systematic literature search was performed to identify articles published from January 2000 to August 2016. Data were pooled for meta-analysis using Review Manager 5.3.

**Conclusions:**

Histopathologic tumor invasion of the SMV/PV is associated with more aggressive biologic behavior and could be used as an indicator of poor prognosis after PDAC resection.

## INTRODUCTION

Pancreatic ductal adenocarcinoma (PDAC) ranks as the fourth leading cause of cancer-associated death in the United States and leads to an estimated 227,000 deaths per year worldwide [1]. Complete resection is the most effective modality for improving the survival of PDAC patients, with an estimated 5-year survival rate of 4–25% [2]. Due to the anatomical proximity, direct tumor infiltration of the superior mesenteric vein (SMV)/portal vein (PV) is not uncommon in PDAC. In an attempt to obtain a negative surgical margin (R0 resection), pancreatectomy with SMV/PV resection is often necessary in these patients. Histopathologic tumor invasion was detected in approximately 21–100% SMV/PV specimens resected [3]. The impact of histopathologic tumor invasion of the SMV/PV on disease prognosis in PDAC remains controversial at present [4–10]. Several studies reported that patients with histopathologic tumor invasion of the SMV/PV had worse survival than those without venous invasion [4, 6], while others failed to demonstrate a significant difference [5, 7–10]. The aim of the present meta-analysis is to assess the prognostic value of histopathologic tumor invasion of the SMV/PV in PDAC.

## RESULTS

### Selection of studies

**Figure 1:**
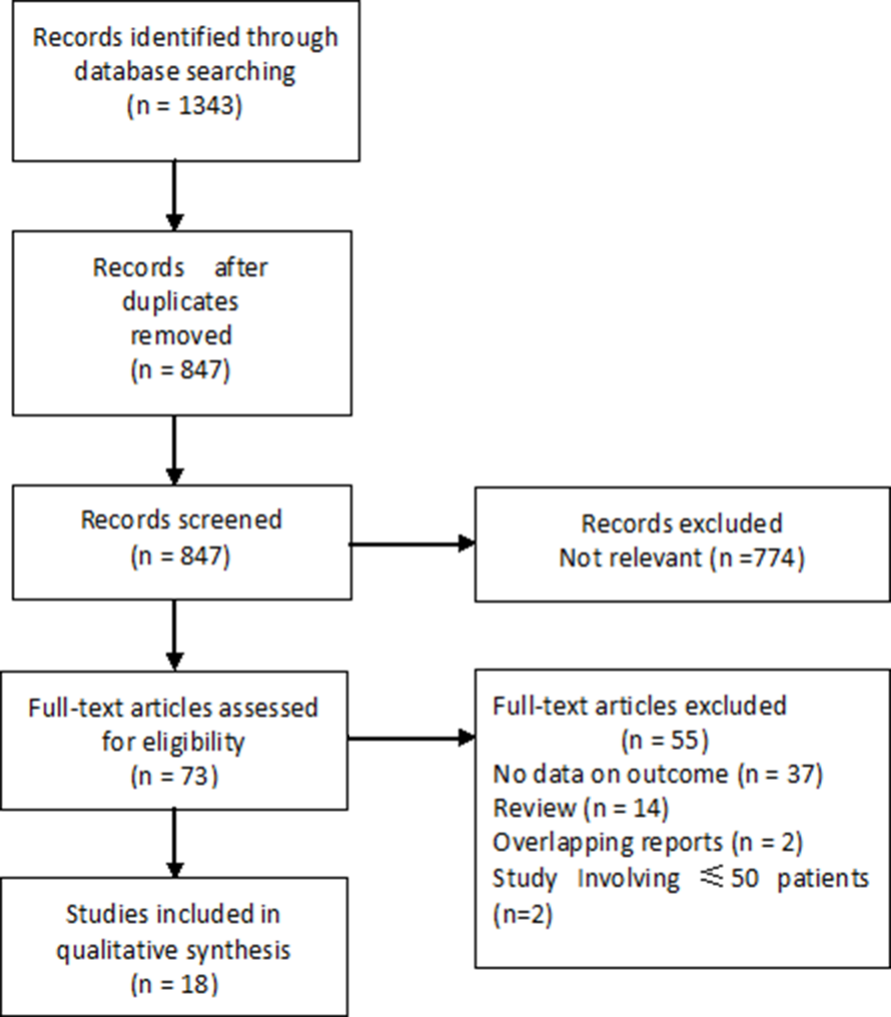
Flowchart of the study selection

A systematic search yielded 18 retrospective studies involving a total of 5242 patients fulfilling the eligibility criteria (Figure 1). The characteristics of the 18 studies included in this meta-analysis are presented in Table 1 [4–21]. Of them, 2199 (41.9%; range 17–100%) patients received SMV/PV resection. Of the 2199 patients receiving SMV/PV resection, pathologic analysis regarding the presence or absence of tumor invasion of the venous wall was available in 2096 patients, in whom 1218 (58.1%) patients had histopathologic evidence of SMV/PV invasion, while no true tumor infiltration was observed in the remaining 878 (41.9 %) patients.

**Table 1:** Study population characteristics of included studies

Author (year)	Country	No. of patients	SP PD/DP/TP	SMV/PV R *n* (%)	Histologic VI *n* (%)	R0 resection *n* (%)	Mortality *n* (%)	5-year OS (%)
Hartel (2002) [4]	Germany	271	243/0/28	68 (25)	56 (82)	191 (70)	9 (3.3)	17
Capussotti (2003) [5]	Italy	100	100/0/0	22 (22)	18 (82)	20/30 (66.7)	NA	8.4
Nakagohri (2003) [6]	Japan	81	61/20/0	33 (41)	17 (51.5)	18 (22)	6 (7.4)	8.5
Poon (2004) [7]	Hong Kong	50	50/0/0	12 (24)	6 (50)	43 (86)	1 (2)	NA
Riediger (2006) [8]	Germany	110	110/0/0	36 (32.7)	14/26 (53.8)	NA	NA	15
Shimada (2006) [9]	Japan	149	143/0/6	86 (58)	58 (67%))	107 (72)	1 (1)	27
Yekebas (2008) [10]	Germany	482	NA	100 (21)	77 (77)	403 (83.6)	23 (3.9)	NA
Ouaissi (2010) [11]	Belgium	149	136/0/13	59 (39.6)	24 (40.6)	109 (73.2)	3 (2)	19.3
Han (2012) [12]	Korea	60	56/0/4	19 (31.7)	15 (78.9)	60 (100)	2 (3.3)	23
Wang (2012) [13]	USA	225	225/0/0	85 (38)	57 (67)	198 (88)	NA	32.2 a
Wang (2014) [14]	Australia	122	122/0/0	64 (53)	47/62 (75.8)	83 (68)	0 (0)	25
Delpero (2015) [15]	France	1399	1325/0/74	402 (30)	173 /311 (56)	1045 (76)	53 (4)	26 a
Jeong (2015) [16]	Korea	276	276/0/0	46 (17)	30 (65.2)	226 (82)	3 (1)	NA
Murakami (2015) [17]	Japan	937	937/0/0	435 (46)	259 (60)	693 (74.1)	19 (2)	21.2
Okabayashi (2015) [18]	Japan	160	105/55	160 (100)	62 (38.7)	93 (58.1)	0 (0)	31.6
Lapshyn (2016) [19]	Germany	86	860/0	86 (100)	39 (45.3)	61 (71)	0 (0)	9
Mierke (2016) [20]	Germany	179	NA	113 (63.1)	36 (31.9)	124 (69.3)	7 (3.9)	18.2 a
Ramacciato (2016) [21]	Italy	406	301/87/18	406 (100)	230 (56.7)	NA	29 (7.1)	24.4

Abbreviations: SP = surgical procedures; PD = pancreaticoduodenectomy; DP = distal pancreatectomy; VI = venous invasion; OS = overall survival;

SMV/PV R = superior mesenteric vein/portal vein resection; NA = not available; a Median;

### Meta-analysis

Seven studies reported comparison of the clinicopathologic features between patients with and without histopathologic SMV/PV invasion [4, 7, 10, 13, 18–20]. Pooled analysis showed that patients with SMV/PV invasion had higher rates of poor tumor differentiation (*P* = 0.002), lymph node metastasis (*P* < 0.001), perineural invasion (*P* < 0.001), positive resection margins (*P* = 0.004), and postoperative tumor recurrence (*P* < 0.001) as compared with patients without SMV/PV invasion, including those who underwent pancreatectomy without SMV/PV resection (Table 2).

**Table 2:** Results of the meta-analysis on clinicopathologic features

Outcome of interest	No. of studies	Results HVI No HVI	Odds ratio	95% CI	*P*-value	I^2^ (%)
Poor tumor differentiation	7	38.9% 32.6%	1.51	1.16, 1.97	0.002	12
lymph node metastases	6	83.3% 71.8%	2.28	1.63, 3.19	< 0.001	15
Lymph node ratio ≥ 0.1	2	58.6% 53.7%	1.28	0.73, 2.24	0.39	36
Perineural invasion	5	65.7% 49.6%	2.23	1.50, 3.30	< 0.001	34
Positive resection margin	7	33.1% 18.6%	2.28	1.30, 4.00	0.004	70
Recurrence	4	85.5% 64.3%	3.39	2.11, 5.43	< 0.001	0

Abbreviations: CI = confidence interval, HVI = histopathologic venous invasion.

**Figure 2:**
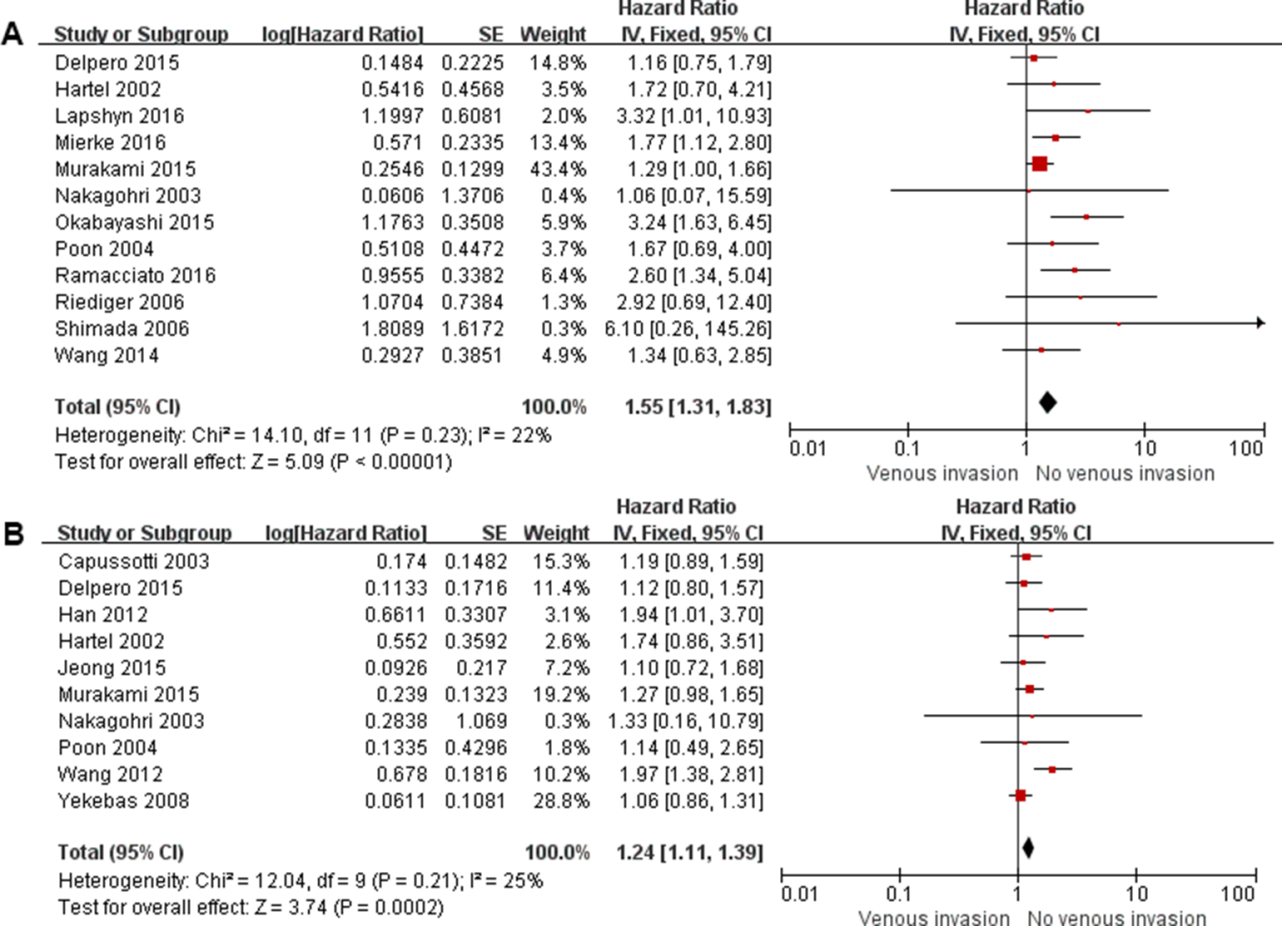
Forest plot for the impact of venous invasion on overall survival in total patients (**A**) and in patients with SMV/PV resection (**B**).

The impact of SMV/PV invasion on overall survival (OS) in total patients who underwent pancreatectomy with and without SMV/PV resection and in patients who underwent pancreatectomy with SMV/PV resection was evaluated in 10 [4–7, 10, 11–13, 15–17] and 12 [4, 6, 7–9, 15, 17–21] studies respectively. The pooled hazard ratio (HR) was 1.24 (95% confidence intervals [CI]: 1.11–1.39; *P* < 0.001) and 1.55 (95% CI: 1.31–1.83; *P* < 0.001) respectively (Figure 2). There was no evidence of heterogeneity in these comparisons. In sensitivity analysis, removal of any single study from the analysis did not change the results significantly (data not shown). Also, the results from four subgroup analysis are similar to those from overall analysis (Table 3).

**Table 3:** Subgroup analysis for the influence of venous invasion on prognosis after pancreatic ductal adenocarcinoma resection

Subgroup	No. of studies	HR	95% CI	*P*-value	I^2^ (%)
Total patients					
Patients underwent PD	5	1.32	1.13, 1.55	< 0.001	36
Unadjusted HR	6	1.58	1.26, 1.98	< 0.001	8
Multivariable adjusted HR	5	1.26	1.04, 1.52	0.02	55
Studies with > 100 cases	7	1.23	1.09, 1.38	< 0.001	41
Patients with SMV/PV R					
Patients underwent PD	5	1.39	1.11, 1.74	0.004	0
Unadjusted HR	8	2.11	1.60, 2.78	< 0.001	0
Multivariable adjusted HR	5	1.44	1.20, 1.74	< 0.001	46
Studies with > 100 cases	5	1.70	1.21, 2.38	0.002	62

Abbreviations: CI = confidence interval; HR = hazard ratio; PD = pancreaticoduodenectomy;

SMV/PV R = superior mesenteric vein/portal vein resection.

Only two studies reported disease-free survival (DFS) in total patients [11, 13]. Multivariable analysis was performed for all two studies. This combined analysis of two studies indicated that patients with SMV/PV invasion had a significantly shorter DFS (HR: 1.82, 95% CI, 1.34–2.48; *P* < 0.001) with no heterogeneity (I^2^ = 0%). Sensitivity analysis and subgroup analysis were not performed due to small number of studies.

### Publication bias

**Figure 3:**
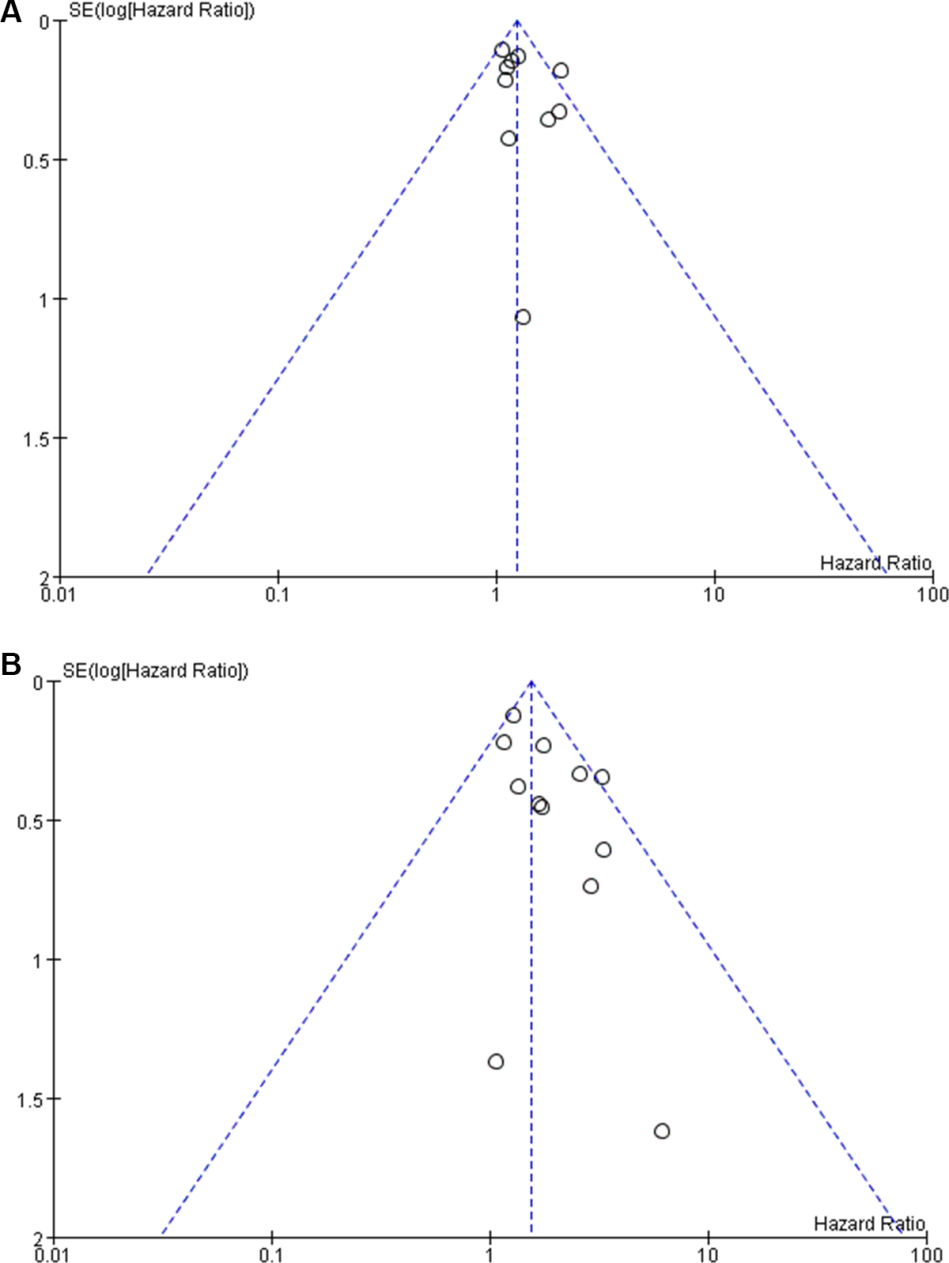
Funnel plot for the impact of venous invasion on overall survival in total patients (**A**) and in patients with SMV/PV resection (**B**).

Funnel plots demonstrated that the impact of SMV/PV invasion on OS was symmetric in total patients and in patients with SMV/PV resection, suggesting the absence of publication bias (Figure 3).

## DISCUSSION

The present study systematically reviewed the literature available and found that histopathologic tumor invasion of the SMV/PV is a poor prognostic indicator in PDAC patients who underwent pancreatectomy. SMV/PV invasion was strongly associated with higher rates of poor tumor differentiation, lymph node metastasis, perineural invasion, positive resection margins, and postoperative tumor recurrence, indicating that PDAC with SMV/PV invasion has more aggressive biological behavior that contributes to poorer outcomes.

Accurate diagnosis of the presence or absence of SMV/PV invasion is primarily important for decision making of an appropriate surgical approach for PDAC resection. However, it is usually difficult to assess SMV/PV wall invasion preoperatively by imaging technologies available at present. Even during operation, it is also difficult to differentiate between “true” invasion and dense inflammatory adhesions caused by peritumoral inflammatory change [10]. SMV/PV narrowing can be classified as type A (no narrowing), B (unilateral narrowing), C (bilateral narrowing), or D (stenosis or obstruction with collaterals), according to preoperative findings on the portal phase of superior mesenteric angiography or intraoperative portography [25]. Nakao and colleagues compared this classification with careful post operative histological examination of the resected vein in 89 of the 101 PDAC patients who underwent pancreatectomy [25]. Histologic evidence of carcinoma invasion into the SMV/PV wall was confirmed in 22 type A cases (100%), 20 type B cases (80%), 8 type C cases (29.6%), and 2 type D cases (7.4%). The survival rates in these patients were higher than those in patients who did not undergo resection. The survival rate of patients with type A, B and C invasion was significantly higher than that of patients with type D invasion. However, the survival rates of patients who did not undergo resection and patients with type D invasion were comparable, indicating that angiographic classification may help select appropriate candidates who are likely to benefit from SMV/PV resection.

As histological SMV/PV invasion is associated with an increased rate of incomplete tumor resection, aggressive local therapy seems sagacious for the sake of radical resection. Recently, irreversible electroporation, a high-voltage, short-pulse, cellular energy ablation device has been proposed as a method to augment/accentuate the margin during PDAC resection [26], though further investigations are needed to reinforce these preliminary data in a large number of cases.

The role of neoadjuvant therapy in patients with vein involvement is a matter of debate [24]. In a study involving 492 PDAC patients who underwent pancreaticoduodenectomy without neoadjuvant therapy, Kelly *et al*. [27] reported that there was no significant difference in R0 resection and DFS or OS between the 70 (14%) patients who received SMV/PV resection and 422 (86%) patients who did not receive SMV/PV resection. They therefore concluded that neoadjuvant therapy was not indicated for patients with vein involvement. However, they did not provide information on histological evidence of true venous involvement. Ferrone *et al*. [28] reported a R0 resection rate of 92% in their single-institutional study on neoadjuvant therapy in a cohort of 40 patients with locally advanced or borderline resectable PDAC. In addition, they found that OS was increased significantly, and lymph node positivity or perineural invasion was decreased significantly in patients receiving neoadjuvant therapy as compared with those in patients without receiving neoadjuvant therapy (35% vs. 79% and 72% vs. 95% respectively). In this context, neoadjuvant treatment may be justified in cases with preoperative suspicion of SMV/PV invasion. Traditionally, gemcitabine- or 5-fluorouracil (5FU)-based protocols are mainly used regimens of neoadjuvant therapy. Recently, the combination of 5FU + oxaliplatin + irinotecan + leucovorin (FOLFIRINOX) has emerged as an alternative in the neoadjuvant setting [29]. Randomized controlled trials are necessary to compare these regimens.

Our study has some limitations. First, although numerous studies have investigated the oncologic outcomes after synchronous SMV/PV resection, not all studies differentiated between true SMV/PV invasion and peritumoral inflammation. Thus, the significant effect of the histopathologic tumor invasion of the SMV/PV on prognosis was underestimated. Second, all included studies were observational in nature, introducing a substantial risk of bias. The reported incidence of histopathologic tumor invasion of the SMV/PV varies widely among studies ranging from 31.9% to 82%, probably reflecting difference in patient selection criteria. Third, there is a question that the poor outcome may be attributed to other unfavorable prognostic factors associated with SMV/PV invasion, unless otherwise further confirmed by a multivariate model. Indeed, the results of our pooled data of multivariate HR are similar to the findings from overall analysis, indicating that SMV/PV invasion has important independent prognostic significance. Finally, because of the limited and heterogeneous patient groups, the significance of the depth of the SMV/PV wall invasion cannot be analyzed.

In conclusion, the present meta-analysis demonstrated that histopathologic tumor invasion of the SMV/PV has more aggressive biological behavior and could be used as an indicator of poor prognosis after PDAC resection.

## MATERIALS AND METHODS

### Study selection

The present study was performed by following the recommendations of the Preferred Reporting Items for Systematic Reviews and Meta-Analyses (PRISMA) Statement [22]. Medline and EMBASE databases were searched from January 2000 to August 2016. Medical subject heading major topic “pancreatic neoplasm,” and the search terms “pancreatic cancer,’’ “portal vein,” and “superior mesenteric vein,” were used in combination with the Boolean operators AND or OR. Bibliographies of the retrieved studies were manually searched for additional studies.

### Criteria for inclusion and exclusion

For inclusion in the meta-analysis, a study had to report on the impact of histopathologic tumor invasion of the SMV/PV on the long-term outcome of PDAC patients who underwent pancreatectomy. Abstracts, letters, editorials and expert opinions, reviews without original data, case reports, nonhuman studies, non-English language studies, studies with fewer than 50 patients, and studies that included the whole set of periampullary lesions (duodenal, ampullary, and biliary) in the same study cohort without separate assessments were excluded.

### Data extraction and outcome measure

Two reviewers (Ailin Song and Farong Liu) independently reviewed each study using standardized data extraction forms. Parameters extracted included first author, year of publication, the country in which the study was performed, study design, inclusion and exclusion criteria, patient characteristics, and all available long-term outcomes. Disagreement was resolved by discussion and consensus.

The primary outcome measure was OS and DFS. Secondary outcome was clinicopathologic features.

### Assessment of methodological quality

The methodological quality of the included studies was assessed by using the Newcastle-Ottawa Scale. Scores were assigned for patient selection, comparability of the study groups, and outcome assessment [23].

### Statistical methods

The effect measures estimated were odds ratios (OR) with 95% CI for dichotomous variables. The HR with 95% CI was used to assess the prognostic value of venous invasion, where an observed HR > 1 implied a worse survival for venous invasion group. For studies without providing the HR and CI, they were calculated from original papers according to the methods described by Parmar *et al*. [24]. To assess heterogeneity across studies, the I^2^ statistic was calculated and a value > 50% was interpreted as statistically significant. A funnel plot based on the OS outcome was conducted to explore the possibility of publication bias. Statistical analyses were performed with Review Manager 5.3 (The Cochrane Collaboration, Software Update, Oxford). A value of *P* < 0.05 was considered statistically significant.

## Abbreviations

SMV: superior mesenteric vein
PV: portal vein
PDAC: pancreatic ductal adenocarcinoma
CI: 95% confidence interval
OR: odds ratios
HR: hazard ratios
OS: overall survival
NOS: Newcastle-ottawa Scale
PRISMA: the Preferred Reporting Items for Systematic Reviews and Meta-Analyses
